# Eukaryotic Initiation Factor 4E (eIF4E) and angiogenesis: prognostic markers for breast cancer

**DOI:** 10.1186/1471-2407-6-231

**Published:** 2006-09-30

**Authors:** Sheng Zhou, Guo-Ping Wang, Cong Liu, Muxiang Zhou

**Affiliations:** 1 Department of Pathology, Tongji Hospital, Tongji Medical College, Huazhong University of Science and Technology, Wuhan, Hubei, P.R. China; 2 Division of Pediatric Hematology/Oncology, Aflac Cancer Center and Blood Disorders Service, Department of Pediatrics, Emory University School of Medicine, Atlanta, Georgia, USA

## Abstract

**Background:**

The overexpression of eukaryotic translation initiation factor 4E (eIF4E), a key regulator of protein synthesis, is involved in the malignant progression of human breast cancer. This study investigates the relationship between eIF4E and angiogenesis, as well as their prognostic impact in patients with human breast cancer.

**Methods:**

Immunohistochemical staining was used to determine protein expression of eIF4E, vascular endothelial growth factor (VEGF), interleukin-8 (IL-8), and CD105 in a set of 122 formalin-fixed, paraffin-embedded primary breast cancer tissues. Expression of eIF4E in positive cells was characterized by cytoplasmic staining. Evaluation of VEGF and IL-8 in the same tissue established the angiogenic profiles, while CD105 was used as an indicator of microvessel density (MVD).

**Results:**

A significant relationship was found between the level of eIF4E expression and histological grade (*P *= 0.016). VEGF, IL-8, and MVD were closely related to tumor grade (*P *= 0.003, *P *= 0.022, and *P *< 0.001, respectively) and clinical stage (*P *= 0.007, *P *= 0.048, and *P *< 0.001, respectively). Expression of eIF4E was also significantly correlated with VEGF (*P *= 0.007), IL-8 (*P *= 0.007), and MVD (*P *= 0.006). Patients overexpressing eIF4E had significantly worse overall (*P *= 0.01) and disease-free survival (*P *= 0.006). When eIF4E, histological grade, tumor stage, ER, PR, Her-2 status and the levels of VEGF, IL-8, MVD were included in a multivariate Cox regression analysis, eIF4E emerged as an independent prognostic factor for breast cancer (*P *= 0.001), along with stage (*P *= 0.005), node status (*P *= 0.046), and MVD (*P *= 0.004).

**Conclusion:**

These results suggest that higher eIF4E expression correlates with both angiogenesis and vascular invasion of cancer cells, and could therefore serve as a useful histological predictor for less favorable outcome in breast cancer patients, as well as represent a potential therapeutic target.

## Background

Angiogenesis, which is essential for both tumor growth and metastasis, depends on the production of angiogenic factors by tumor cells and normal cells. Several pathways are involved, including those involved with the secretion of angiogenic substances, activation of endothelial cells, degradation of capillary membranes, and endothelial cell migration. The resulting increased vascularity enhances the growth of the primary neoplasm and provides a greater chance for hematogenous metastasis [1]. At the beginning of this undesirable angiogenic process, two potent angiogenic growth factors, VEGF and IL-8, are known to stimulate endothelial cell proliferation, induce microvessel permeability, and begin to establish the tumor neovasculature [2]. Disruption of the capacity to produce or upregulate these factors should help to control the progression of cancer.

VEGF, a well-known contributor to angiogenic processes in breast cancer, exhibits an increase in expression during pre-invasive cancer progression and is significantly associated with high intratumoral microvessel density [3-6]. The level of VEGF expression independently predicts disease-free survival (DFS) and overall survival (OS) in invasive breast carcinoma [7,8].

IL-8, a CXC chemokine originally identified as a neutrophil chemotactic factor, was subsequently recognized to have many functions that promote tumor growth, angiogenesis, and metastasis [9]. It is released by both stromal cells and tumor-associated macrophages in the tumor microenvironment, and is considered to have pro-angiogenic and pro-malignant effects [10]. Recently, it has been shown that an elevated level of IL-8 expression in breast cancer is accompanied by a high level of VEGF expression [11]. In addition, high level of IL-8 expression is associated with the invasive potential of breast cancer cells [12].

Up-regulation of angiogenic growth factor synthesis is directly related to mediation of transcriptional and translational events. One of the major translation factors, eIF4E, plays a key role in cellular protein synthesis. It is a 25 kD protein that recognizes the 7-methylguanosine-containing cap at the 5-prime terminus of mRNA, and it assists in the transfer of mRNA to the 48S ribosomal complex. By binding to this cap, eIF4E facilitates the attachment of the "RNA helicase complex," known as eIF4F [13]. A number of observations suggest that increased expression of eIF4E might be one of the key elements leading to angiogenesis, which ultimately results in tumor metastasis [14]. Recently, McClusky et al. suggested that eIF4E is strongly involved in increasing the risk for tumor recurrence and in a poor prognosis for patients with node-positive breast cancer [15]. In the present study, we have examined this possibility as well as furthered the investigation into eIF4E's relationship to angiogenesis, by testing biopsies from a large sample of patients with untreated breast cancer.

Our findings suggest that eIF4E expression is closely related to IL-8 and VEGF expression in breast cancer cells, possibly contributing to disease progression. We strongly believe that identification of mediators of translation such as eIF4E may enable future design of therapeutic modalities that can jointly target several pro-malignancy factors at once. Histological testing for these factors would indeed be useful in assessing patient risk, but translation of this knowledge into therapy would be even more useful. Implementation of a therapeutic approach that includes control of angiogenesis is more likely to lead to improved treatment outcomes for all breast cancer patients, including those at higher risk.

## Methods

### The collection of tissue samples from breast cancer patients

Tissue samples were studied from 122 breast carcinoma patients ranging from 41 to 77 years of age (median age, 56.2 years), who had not received chemotherapy, radiotherapy, or hormone therapy prior to surgery. All patients were treated at the Department of Surgery in Tongji Hospital (Huazhong University of Science and Technology, Wuhan, China) and were sequential cases seen from October 1996 to July 1999. These select untreated breast cancer patients underwent axillary node excision combined with wide local excision with margin clearance or mastectomy. Study samples were collected at this time. Adjuvant axillary irradiation, systemic chemotherapy, and antiestrogen therapy were then offered and administered as indicated per current post-surgical standard of care. Patient follow-up ranged from 1–72 months (median, 44 months): Clinical records show that 77 patients were found to be disease-free, while 45 breast cancer patients relapsed, of whom 30 subsequently died. The 5-year survival rate was 76.4%. Mean survival time was 41.89 ± 8.38 months and median survival time was 39 months. Overall patient survival was calculated as the period from surgery until the date of death, while disease-free survival was the period from surgery to date of metastasis.

The research protocol was approved by our Institutional Review Board (IRB) before all specimens were examined by experienced pathologists. Histological examination was carried out on paraffin-embedded sections stained with hematoxylin & eosin (H&E). These specimens had a spectrum of breast carcinomas represented, including 80 invasive ductal carcinomas (IDC), 15 ductal carcinomas *in situ *(DCIS), and 5 lobular carcinomas *in situ *(LCIS). In addition, special invasive carcinoma types included: 14 invasive lobular carcinomas (ILC), 6 medullary carcinomas (MC), and 2 tubular carcinomas (TC). The 102 invasive carcinoma cases seen included 27 welldifferentiated (grade I), 41 moderately differentiated (grade II), and 34 poorly differentiated (grade III) carcinomas. Similarly, there were 5 low-grade, 3 intermediate-grade, and 12 high-grade cases in the non-invasive carcinomas (DCIS and LCIS). Tumor size varied from 0.5 to 6.8 cm (mean, 3.1 cm). At the time of surgery, it was found that a total of 74 patients had experienced lymph node metastases. Other clinical and pathologic parameters were obtained from the hospital pathology reports, including estrogen receptor (ER) and progesterone receptor (PR) status, Her2/neu status, and the TNM stage (Table 1).

### Immunohistochemistry for eIF4E

The paraffin-embedded, formalin-fixed archival breast tissue was cut into four-micrometer sections and fixed on charged slides using heat immobilization, by baking at 60°C for 2 hours. The slides were then deparaffinized in xylene and subsequently rehydrated through a graded ethanol series ending in deionized water. Any endogenous peroxidase was blocked by treatment with 3% hydrogen peroxide for 15 minutes, followed by 3 rinses of 5 minutes each in deionized water. Antigen retrieval was performed by placing slides in 1× citrate buffer for 15 minutes at 100°C (in a microwave). The eIF4E primary antibody (Santa Cruz, California, USA), diluted to 1:100 in phosphate-buffered saline (PBS) and 1% bovine serum albumin, was left on tissue overnight at 4°C, in a humidified chamber. As a negative control, preimmune serum was substituted for the primary antibody. All sections were rinsed in PBS 3 times, 5 minutes each. Secondary antibody (Envison, Anti-Mouse/Rabbit-HRP, DAKO) was applied to all sections and incubated for 30 minutes at room temperature (RT), followed by 3 PBS rinses of 5 minutes each. Finally, the sections were developed with 3,3'-diaminobenzidine (DAB) for 5 minutes at RT, then counterstained with hematoxylin. After staining, sections were dehydrated through a graded series of ethanol baths followed by xylene and cover-slipped. Using microscopy, the section's tumor cell immunoreactivity for eIF4E was scored according to the cytoplasmic staining present: Both the percentage of positively stained tumor cells and the stain intensity were taken into account to determine eIF4E expression values. The percentage of positive cells was rated as follows: 1 point, <5% positive tumor cells; 2 points, 5–25% positive cells; 3 points, 26–75% positive cells; and 4 points, >75% positive cells. Stain intensity was rated as follows: 1 point, weak intensity; 2 points, moderate intensity; and 3 points, strong intensity. The breast cancer specimens were attributed to four groups, according to their overall score: Absent expression, when <5% of cells stained positive, regardless of intensity; weak expression, a total of 3 points; moderate expression, 4–5 points; and strong expression, 6–7 points. For statistical reasons, tumors were then scored according to a two-scale system: Low reactivity denoting tumors with absent or weak expression, and high reactivity denoting tumors with moderate to strong expression. The association of eIF4E with other parameters was assessed using eIF4E as either a categorical variable (low reactivity vs. high reactivity) or a continuous variable (the percentage of eIF4E-positive cells within a sample).

### VEGF and IL-8 detection and assessment

VEGF and IL-8 expression were assessed using purified mouse anti-human monoclonal antibodies (purchased from Santa Cruz, California, USA), diluted 1:100 and 1:50, respectively, in an overnight incubation, following standard immunohistochemistry procedures. The percentage of cancer cells having cytoplasmic staining for VEGF or IL-8 reactivity was recorded. Staining was graded in a four-grade classification as follows: -, for those where reactivity was not detected; 1+, those with positive stain in less than 5% of tumor cells; 2+, a positive stain between 5% and 50%; and 3+, when greater than 50% positive. The median value was used as a cut-off point to define those tumors having either high or low reactivity for VEGF and IL-8.

### Evaluation of microvessel density

Tumor angiogenesis was assessed by calculating standard microvessel density (MVD). Microvessels were identified by immunostaining endothelial cells with the mouse anti-human monoclonal antibody CD105, which targets the receptor for the transforming growth factor beta 1 (TGFβ-1). Although the TGFβ-1 receptor has weak or negative expression in the vascular endothelium of normal human tissues, it is more specific and sensitive in the vascular endothelium of cancer tissues [16]. First, each slide was scanned at low magnification (×100) to identify four areas with the highest density of microvessels ("hotspots"). Each hotspot was then evaluated at high power magnification (×400) for the number of stained microvessels per field. Any endothelial cell having a brown stain was considered to be a single countable microvessel, and the presence of a clearly defined vessel lumen was not required to verify the structure. The final microvessel score was the average of vessel counts from four fields. MVD was quoted as a continuous variable.

### Statistical analysis

One-way ANOVA testing was used to assess correlations between the continuous variables. Contingency tables were analyzed for trends with the chi-square test, in order to evaluate differences in clinicopathologic factors between the patient groups. The Spearman rank correlation coefficient test was used to examine the correlations between different variables. Linear regression analysis was also used to assess the correlation between continuous variables. Survival curves were plotted using the method of Kaplan and Meier and the significance of the observed differences was assessed using the log-rank test. Survival analysis was carried out using either cancer recurrence or death from disease as the endpoints for disease-free survival (DFS) and overall survival (OS), respectively. The influence of each variable on survival was assessed with the Cox proportional hazards regression model. The level of statistical significance was defined as P < 0.05. All analyses were carried out using SPSS 11.0 for Windows 2000 software.

## Results

### Immunochemical staining of eIF4E, VEGF, IL-8, and CD105 in breast cancer tissue

**Figure 1:**
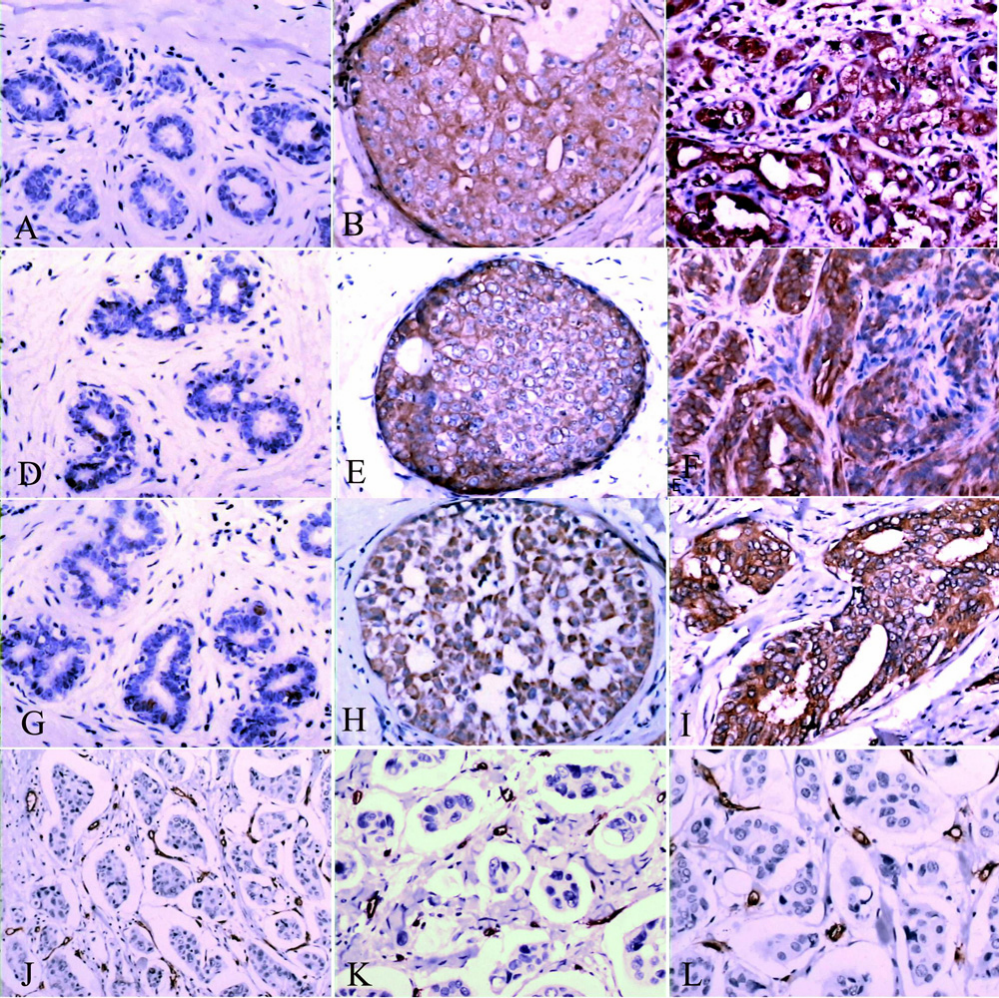
Representatives of immunohistochemical staining for eIF4E, VEGF, IL-8 and CD105 expression in breast cancer. A, slide for eIF4E expression in the adjacent normal ductal epithelial cells (negative), B, eIF4E expression in the cytoplasm of tumor cells of ductal carcinomas *in situ *(moderate positive), C, eIF4E expression in invasive ductal carcinoma cells (strong positive); D-F and G-I, representative panels for VEGF and IL-8 expression, respectively, in 3 types of tissues as detected for eIF4E expression in (A-C); J-L, representative slides for positive CD105 expression in 3 breast cancer specimens with invasive ductal carcinoma (×200).

In breast carcinoma, localization of eIF4E protein overexpression was mainly cytoplasmic in the cancer cells, but not in the adjacent normal-looking ductal epithelial cells and stromal elements. Of 122 tumors, 59 (48.4%) showed low reactivity (absent or weak expression) and 63 (51.6%) showed high reactivity (moderate to strong expression) (Fig. 1A,B, and 1C). Immunopositive reactions for VEGF (Fig. 1D,E and 1F) and IL-8 (Fig. 1G,H and 1I) were confined to the cytoplasm of cancer cells. Expression of VEGF was found to be negative, weak, moderate, and strong in 29 (23.8%), 38 (31.1%), 34 (27.9%), and 21 (17.2%) tumor tissues, respectively. Also, the IL-8 protein showed negative, weak, moderate, and strong staining in 21 (17.2%), 40 (32.8%), 37 (30.3%) and 24 (19.7%) of 122 tumors, respectively. In addition, it was evident that both VEGF and IL-8 had higher immunoreactivity in the IDC than in the DCIS. In a few specimens, the most intense reaction was demonstrated distal to the blood vessels and surrounding necrotic areas, but in the vast majority of positive cancer cells we observed a diffuse expression pattern that was independent of vessel proximity.

Vascular endothelial cell or endothelial cell clustering around the tumors was evident using the anti-CD105 antibody (Fig. 1J,K and 1L). The mean value of MVD was found to be 15.22 ± 6.93. The microvessels around the solid tumor nest were much denser than those in the adjacent normal tissues.

### Correlation of eIF4E, VEGF, IL-8, and MVD with clinicopathologic parameters

A significant positive association between eIF4E immunoreactivity and the histological grade of the invasive tumor was found (*p *= 0.016). In addition, we found that invasive carcinoma was different from non-invasive carcinoma with respect to eIF4E immunostaining (*p *= 0.034). No significant correlations occurred between eIF4E expression and patients' age (*p *= 0.49), histological type in invasive carcinoma (*p *= 0.72), tumor size (*p *= 0.25), node metastasis (*p *= 0.23), TNM stage (*p *= 0.54), or the ER, PR, and HER-2 status provided (*p *= 0.44, *p *= 0.38, and *p *= 0.45, respectively) (Table 2). In addition, we found that VEGF and IL-8 were closely related to both the tumor grade (*p *= 0.003 and *p *= 0.022, respectively) and the clinical stage (*p *= 0.007 and *p *= 0.048, respectively) (Table 3). Increasing MVD was also linked with increasing tumor grade (*p *< 0.001) and stage (*p *< 0.001) (Table 4).

### Correlation of eIF4E with angiogenesis markers

**Figure 2:**
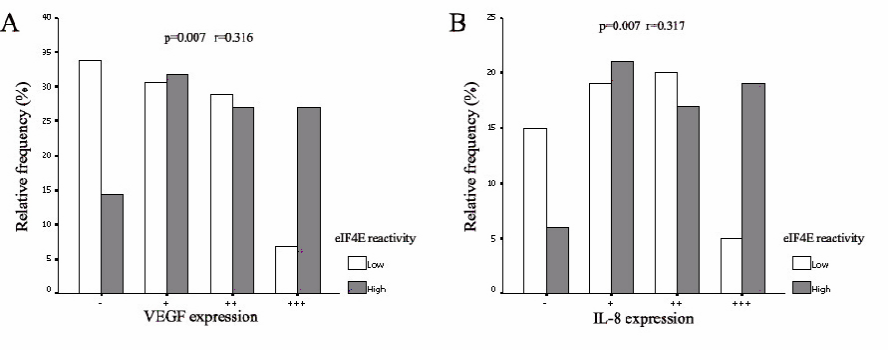
Graphical representation for analysis of the correlation between eIF4E expression and VEGF levels (A) as well as with IL8 expression (B) in breast cancer.

**Figure 3:**
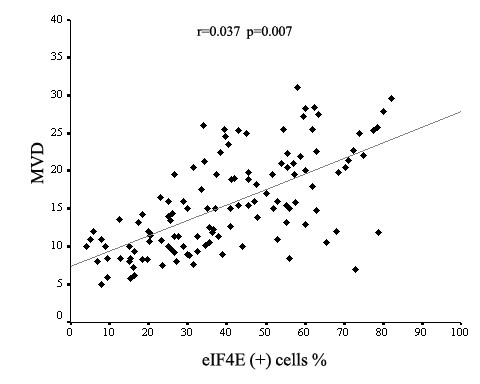
Linear regression analysis of the percentage of eIF4E positively stained cells with MVD.

Using the Spearman rank correlation, significant correlations of eIF4E with both VEGF (*p *= 0.007, r = 0.316) and IL-8 (*p *= 0.007, r = 0.317) expression in breast cancer were noted (Fig. 2A,2B). Using linear regression analysis, we studied the association of the percentage of tumor cells staining positive for eIF4E and MVD. We found a significant correlation between the proportion of eIF4E-positive cells and MVD (*p *= 0.006, r = 0.34) in these breast cancer samples (Fig. 3).

### Survival analysis

**Figure 4:**
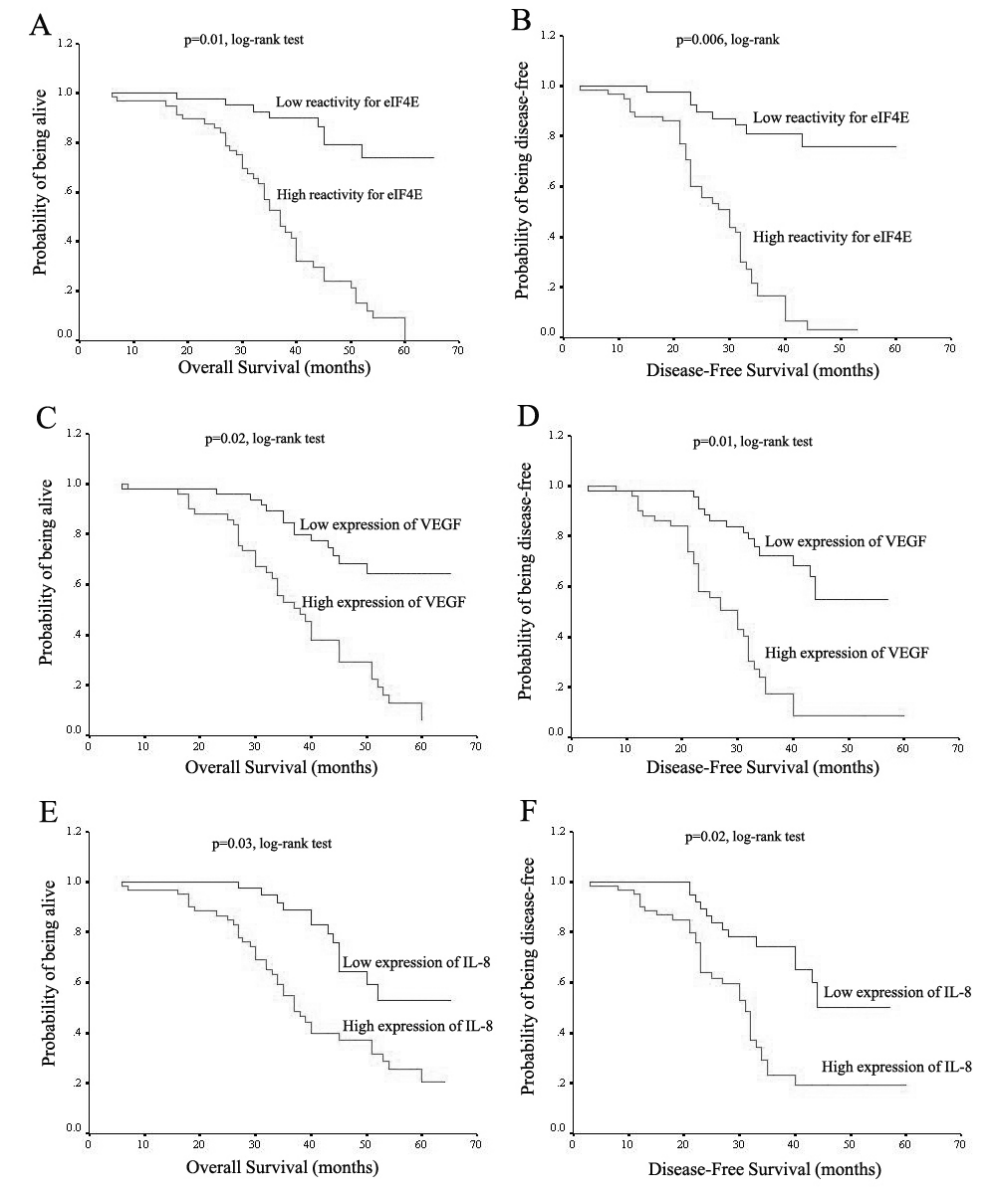
Comparison of overall as well as disease-free survival using the Kaplan-Meier analysis between the groups of patients with low and high eIF4E, VEGF and IL-8 protein expression.

The Kaplan-Meier survival curves for eIF4E, VEGF, and IL-8 (Fig. 4) demonstrated that when the survival of breast cancer patients having moderate to strong expression of eIF4E is compared with the survival of patients with absent to weak expression of eIF4E through log-rank testing, there was a significantly unfavorable influence for eIF4E expression on both the overall survival (OS) (*p *= 0.01) and disease-free survival (DFS) (*p *= 0.006) of the patients (Fig. 4A,B). Univariate analysis revealed that overexpression of VEGF and IL-8 were also associated with a worse prognosis, with both unfavorable OS (*p *= 0.02 and *p *= 0.03, respectively) and DFS (*p *= 0.01 and *p *= 0.02, respectively) linked to these two pro-angiogenic growth factors as well (Fig. 4C–F).

### Multivariate analysis

To examine the independent prognostic significance of eIF4E in relation to the classical clinicopathological parameters and angiogenic variables, multivariate analysis of OS was performed including eIF4E and all three angiogenic markers, as well as other risk factors such as tumor grade, stage and the ER, PR, and Her-2 status (Table 5). In a multivariate model that included tumor grade, stage and the ER, PR, and Her-2 status, as well as levels of VEGF, IL-8, MVD, and eIF4E found in the tissues; eIF4E emerged as an independent prognostic factor for OS (*p *= 0.001), along with stage (*P *= 0.005), node status (*P *= 0.046), and the MVD present (*P *= 0.004). Table 6 shows the results of the multivariate analysis of DFS including the previously mentioned parameters. The eIF4E, MVD, and tumor stage still emerged as independent prognostic factors (*p *= 0.02, *p *= 0.011, and *p *= 0.048, respectively), but node metastasis was found to be of borderline significance (*p *= 0.062).

## Discussion

The initiation of protein translation is now recognized as an important step in the control of gene expression. In eukaryotic cells, most mRNAs are translated through a cap-dependent mechanism of initiation. As a cap-binding protein, eIF4E plays a central role in this process [17,18]. This eIF4E protein was first shown to be overexpressed in breast cancer by Kerekatte et al [19]. Later studies by Li et al [20,21] have demonstrated that the degree of eIF4E overexpression predicts cancer recurrence and outcome in stage I to III breast cancer patients. Cancer specimens exhibiting high levels of eIF4E expression increase the patient's relative risk for cancer recurrence as compared to patient cancer specimens with low eIF4E expression. It has been noted that eIF4E overexpression is associated with tumor angiogenesis in breast cancer [22]. A recent report by Byrnes et al [23] further showed that increasing eIF4E overexpression in breast cancer correlates with MVD counts as well as high VEGF levels. Thus, it appeared that there might be significant relationships between the overexpression of eIF4E, angiogenesis, cancer recurrence, and patient survival in breast cancer.

In the present study, we examine the level of expression of eIF4E in order to analyze any correlations with the molecules initiating angiogenic pathways and patient prognosis, using histological samples from a relatively large number of patients with previously untreated breast cancer. Here we have shown that eIF4E, as revealed by immunohistochemistry, is overexpressed in breast cancer tissue. Indeed, a strong association of eIF4E with an angiogenic profile was observed in these breast cancer patients. Consistent with the observation from the study in patients with stage I to III breast cancer by Byrnes et al [23], our results studied in all stages including stage IV breast cancer patients demonstrated that eIF4E overexpression is associated with high levels of VEGF and MVD counts. In addition, we found that eIF4E overexpression is associated with increased IL-8 expression. Both VEGF and IL-8 expression are up-regulated in breast cancer tissue, most likely triggering the neoangiogenesis process, which is essential for tumor growth and progression. Our observations are indicative of a role for eIF4E as a key regulator of the angiogenic cascade. In support of our premise, we found that both VEGF and IL-8 upregulation are associated with increased MVD.

Furthermore, we also observed that there is an increase in tumor-associated VEGF and IL-8 in patients with unfavorable prognoses. The unfavorable influence of VEGF and MVD on breast cancer outcome has previously been reported [24]. In that study, high VEGF expression and, consequently, enhanced angiogenic effect are found to offer the malignant cells the ability to implant, grow rapidly, and possibly interfere with the immune response among malignant cells, therefore leading to tumor relapse or facilitating invasion and stage progression. We believe that a potential linkage of high eIF4E expression results in the upregulation of angiogenic factors such as VEGF, creating the subsequent increase in tumor MVD and a worse clinical outcome. Our survival analysis for each of the factors we suspect may lead to cancer progression and negative clinical outcomes demonstrated that patients with lower eIF4E expression had a statistically significant lower rate of cancer recurrence, when compared with those patients with higher eIF4E expression (*p *= 0.006, log-rank test). Similarly, we found that patients with higher eIF4E expression had higher mortality than those patients with lower eIF4E expression (*p *= 0.01, log-rank test).

An interesting observation was the fact that when eIF4E was included with grade, stage, MVD, VEGF, and IL-8 expression in the multivariate model, it had an independent negative prognostic effect on overall and disease-free survival (*P *= 0.001 and *P *= 0.02, respectively). Our results did not show correlations of eIF4E expression with nodal status and expression of ER, PR and Her-2/neu. Although nodal status is believed the most important prognosticator for breast cancer outcome, our study indicated a "borderline significance" for overall and disease-free survival (*P *= 0.046 and *P *= 0.062, respectively). In a prospective study designed to specifically address risk for recurrence in patients with node-positive breast cancer, eIF4E overexpression appears to be an independent predictor of a worse outcome independent of nodal status [15]. Based on the previous observation and our results, we hypothesize that eIF4E overexpression is likely more important than nodal status in predicting breast cancer recurrence and outcome.

The overexpression of eIF4E would be responsible for initiating the translation of many polypeptides, including the angiogenic and growth-promoting factors included in this study. Since some of these latter factors would make cancer cells become more proliferative and invasive, our findings may explain the significant association of vascular invasion and a worsened clinical outcome in patients having marked eIF4E expression.

## Conclusion

In conclusion, the results of this study provide evidence that the immunohistochemical level of eIF4E expression is markedly upregulated in high-grade invasive breast cancer tissues. In addition, we found that overexpression of eIF4E might play an important role in tumor progression and microneoangiogenesis. Specifically, our findings show that eIF4E may be an important regulator of production of angiogenic factors, such as IL-8 and VEGF that are associated with poor prognosis in breast cancer. We believe that eIF4E could be a potential target for adjuvant therapy and that it might even substantially enhance the effects of radiation and chemotherapy. The role of eIF4E as a viable target to block the development of angiogenesis and advanced breast cancer deserves further investigation.

## Competing interests

The author(s) declare that they have no competing interests.

## Authors' contributions

SZ carried out the Immunohistochemistry studies, performed the statistical analysis and drafted the manuscript. GW assisted experimental design and manuscript writing. CL participated in the Immunohistochemistry studies. MZ provides concept and reagents. All authors read and approved the final manuscript.

## Pre-publication history

The pre-publication history for this paper can be accessed here:



## Figures and Tables

**Table 1:** Clinical characteristics

Variable	No. of patients (%)
Total	122 (100%)
Age (median, 56 years)	
<56 years	68 (55.7%)
≥56 years	54 (44.3%)
Surgical treatment	
Breast conservation	29 (23.8%)
Mastectomy	93 (76.2%)
Tumor size (mean, 3.1 cm)	
<3.1 cm	70 (57.4%)
≥3.1 cm	52 (42.6%)
Histological diagnosis	
Invasive carcinoma	102 (83.6%)
Ductal	80 (65.6%)
Lobular	14 (11.5%)
Medullary	6 (4.9%)
Tubular	2 (1.6%)
Non invasive carcinoma	20 (16.4%)
DCIS	15 (12.3%)
LCIS	5 (4.1%)
Histological grade	
Invasive carcinoma	102 (83.6%)
Grade I	27 (22.1%)
Grade II	41 (33.6%)
Grade III	34 (27.9%)
Non invasive carcinoma	20 (16.4%)
Low grade	5 (4.1%)
Intermediate grade	3 (2.5%)
High grade	12 (9.8%)
TNM staging	
Stage I	30 (24.6%)
Stage II	33 (27.1%)
Stage III	37 (30.3%)
Stage IV	22 (18.0%)
Node metastatic status	
Negative	48 (39.3%)
Positive	74 (60.7%)
Estrogen Receptor status	
Negative	54 (44.3%)
Positive	68 (55.7%)
Progesterone Receptor status	
Negative	65 (53.3%)
Positive	57 (46.7%)
Her-2/neu status	
Negative	66 (54.1%)
Positive	56 (45.9%)
Clinical outcome	
Disease free	77 (63.1%)
Metastasis or recurrence	45 (36.9%)
Died for breast cancer	30 (24.6%)

**Table 2:** Correlations of eIF4E with clinicopathological parameters

	eIF4E expression					
	
Variable	n	Low reactivity		High reactivity		p value
			
		n	%	n	%	
Total	122	59	48.4	63	51.6	
Age (median, 56 years)						
<56 years	68	31	25.4	37	30.3	0.49
≥ 56 years	54	28	23.0	26	21.3	
Tumor size						
<3.1 cm	70	37	30.3	33	27.0	0.25
≥3.1 cm	52	22	18.1	30	24.6	
Histological diagnosis						
Invasive carcinoma	102	45	36.9	57	46.7	0.034
Non invasive carcinoma	20	14	11.5	6	4.9	
Invasive carcinoma type						
Ductal	80	34	33.3	46	45.1	0.72
Lobular	14	8	7.8	6	5.9	
Medullary	6	2	2.0	4	3.9	
Tubular	2	1	1.0	1	1.0	
Invasive carcinoma grade						
Grade I	27	17	16.7	10	9.8	0.016
Grade II	41	19	18.6	22	21.6	
Grade III	34	9	8.8	25	24.5	
TNM staging						
Stage I	30	16	13.1	14	11.5	0.54
Stage II	33	18	14.8	15	12.3	
Stage III	37	17	13.9	20	16.4	
Stage IV	22	8	6.6	14	11.4	
Node metastatic status						
Negative	48	20	16.4	28	22.9	0.23
Positive	74	39	32.0	35	28.7	
ER status						
Negative	54	24	19.7	30	24.6	0.44
Positive	68	35	28.7	33	27.0	
PR status						
Negative	65	29	23.8	36	29.5	0.38
Positive	57	30	24.6	27	22.1	
Her-2/neu status						
Negative	66	34	27.9	32	26.2	0.45
Positive	56	25	20.5	31	25.4	

**Table 3:** VEGF and IL-8 in relation to clinicopathological indicators and eIF4E reactivity

Variable	VEGF				p value	IL-8				p value
				
	(-)	(1+)	(2+)	(3+)		(-)	(1+)	(2+)	(3+)	
Total	29	38	34	21		21	40	37	24	
Age (median, 56 years)										
<56 years	13	21	17	8	0.43	13	18	24	11	0.24
≥56 years	16	17	27	13		8	22	13	13	
Tumor size										
<3.1 cm	20	20	15	7	0.09	15	20	14	10	0.08
≥3.1 cm	9	18	10	14		6	20	23		
Invasive carcinoma grade										
Grade I	14	19	8	2	0.003	10	17	8	2	0.022
Grade II	10	12	12	6		6	13	11	9	
Grade III	5	7	14	13		5	10	18	13	
TNM staging										
Stage I	10	14	7	1	0.007	9	13	6	3	0.048
Stage II	9	10	9	3		6	14	10	4	
Stage III	7	8	8	4		3	8	9	6	
Stage IV	3	6	10	13		3	5	12	11	
eIF4E expression										
Low reactivity	20	18	17	4	0.007	15	19	20	5	0.007
High reactivity	9	20	17	17		6	21	17	19	

**Table 4:** MVD with tumor grade, stage and eIF4E expression

Variable	n	MVD	p value
		(mean ± S.D.)	(ANOVA)
Total	122	15.22 ± 6.93	
Invasive carcinoma grade			
Grade I	27	10.63 ± 7.54	*p *< 0.001
Grade II	41	14.98 ± 5.72	
Grade III	34	18.36 ± 6.01	
TNM staging			
Stage I	30	10.55 ± 5.36	*p *< 0.001
Stage II	33	12.42 ± 6.17	
Stage III	37	15.62 ± 4.75	
Stage IV	22	25.11 ± 5.62	
eIF4E expression			
Low reactivity	59	10.19 ± 7.02	*p *< 0.001
High reactivity	63	19.93 ± 6.89	

**Table 5:** Cox hazard estimation of OS in breast cancer (Multivariate analyses)

Parameter	Regression Coefficient	Standard Error	Wald	Relative Risk	95% CI		P value
						
					Lower	Upper	
Tumor size	2.749	1.622	2.872	2.572	0.515	2.347	0.098
Node status	-0.807	0.376	4.602	0.446	0.213	0.933	0.046
Stage	0.529	0.189	7.851	1.697	1.172	2.456	0.005
Grade	0.237	0.289	0.118	1.332	0.72	2.232	0.412
ER	0.463	0.489	0.896	1.589	0.609	4.143	0.344
PR	0.226	0.363	0.388	1.253	0.616	2.551	0.533
Her-2	0.263	0.412	0.406	1.301	0.58	2.918	0.524
IL-8	0.383	0.36	1.134	1.467	0.725	2.969	0.287
MVD	0.195	0.068	8.2	1.215	1.063	1.388	0.004
VEGF	0.257	0.411	0.39	1.293	0.578	2.892	0.532
eIF4E	2.232	0.644	12.021	9.316	2.638	32.896	0.001

**Table 6:** Cox hazard estimation of DFS in breast cancer (Multivariate analyses)

Parameter	Regression Coefficient	Standard Error	Wald	Relative Risk	95% CI		P value
						
					Lower	Upper	
Tumor size	1.298	1.572	0.681	3.66	0.277	4.579	0.409
Node status	-0.68	0.365	3.473	0.507	0.248	1.036	0.062
Stage	0.386	0.195	3.908	1.471	1.003	2.156	0.048
Grade	0.145	0.262	0.309	1.157	0.693	1.931	0.578
ER	0.198	0.466	0.181	1.219	0.489	3.037	0.671
PR	0.492	0.35	1.978	1.635	0.824	3.246	0.16
Her-2	0.099	0.39	0.065	1.104	0.514	2.37	0.299
IL-8	0.636	0.381	2.788	1.888	0.895	3.982	0.095
MVD	0.175	0.069	6.479	1.191	1.041	1.363	0.011
VEGF	0.088	0.436	0.041	1.092	0.465	2.564	0.84
eIF4E	1.282	0.549	5.453	3.602	1.229	10.563	0.02
